# Editorial to “Efficacy and safety of pulsed‐field versus conventional thermal ablation for atrial fibrillation: A systematic review and meta‐analysis”

**DOI:** 10.1002/joa3.13138

**Published:** 2024-08-22

**Authors:** Kenji Kuroki, Akira Sato

**Affiliations:** ^1^ Department of Cardiology University of Yamanashi Chuo Yamanashi Japan

**Keywords:** Editorial, Metaanalysis, Pulsed field ablation, Systematic review

We express our gratitude to Amin et al.^1^ for their systematic review and meta‐analysis comparing the efficacy and safety of pulsed field ablation (PFA) versus conventional thermal ablation (TA) for atrial fibrillation (AF). Pulsed field ablation represents an innovative energy source in ablation therapy, employing ultra‐short pulse direct current to induce cell death via electroporation, creating pores in the cell membrane. This method offers several distinct advantages: (1) selectivity for cardiac tissue over other tissues such as nerves, smooth muscle, or red blood cells; (2) effectiveness dependent on electrode proximity to tissue, favoring deep lesions without requiring strong contact force; and (3) nonthermal mechanism, minimizing inflammation and being unaffected by blood flow cooling. These unique features suggest that PFA may offer safer ablation energy compared with TA, potentially enhancing efficacy by enabling more effective energy delivery. Despite accumulating clinical evidence of PFA, most studies remain single‐arm or retrospective with limited sample sizes. Therefore, Amin et al.'s meta‐analysis provides crucial insights into comparing the safety and efficacy of PFA versus TA.

In their study, Amin et al. analyzed 17 studies encompassing 2255 patients, focusing on AF recurrence and all atrial arrhythmia recurrence (AF, atrial tachycardia [AT], and atrial flutter [AFL]) separately during the follow‐up. They found PFA was significantly reduced AF recurrence but did not show a significant difference in all atrial arrhythmia recurrence, potentially indicating higher recurrence of AT or AFL with PFA. Discussions by the authors suggested that extensive PVI using PFA might inadvertently create channels in the left atrial posterior wall, facilitating roof‐dependent atrial tachycardia. Kawamura et al.^2^ demonstrated that there was no significant difference between the PFA and TA cohorts in the nonablated posterior wall area, though the PFA cohort (*n* = 17) had a larger isolation area than radiofrequency ablation cohort (*n* = 17) in the left inferior pulmonary vein in the propensity score‐matched analysis. This potential arrhythmogenic effect warrants further investigation using more larger cohorts.

Regarding complications, Amin et al. observed significantly fewer instances of phrenic nerve palsy and esophageal lesions with PFA, attributed to its tissue selectivity. However, they also noted an increased incidence of pericardial tamponade, which may partly stem from initial operator inexperience with PFA devices. If the rate of tamponade decreases, as more operators become accustomed to PFA devices in the near future, it would prove that operators' inexperience was the true reason. In fact, such a trend has already begun to emerge in a registry trial.^3^ The MANIFEST‐PF registry (*n* = 1568, initial experience of the MANIFEST‐17 K registry), published in 2022, reported a tamponade rate of 0.97%, which is relatively high for experienced institutes. Subsequently, in the MANIFEST‐17 K registry (*n* = 17,642) published in 2023, they reported a dramatic improvement in the tamponade rate to 0.36%. This observation suggests that definitive conclusions should be withheld in a systematic review, especially when comparing a new technology with conventional methods.

Here, we would like to introduce two meta‐analyses to compare PFA and TA, both of which involve a substantial sample size and were published in 2023 and 2024, respectively. De Campos et al.^4^ analyzed 18 studies involving 4998 patients to evaluate the acute and long‐term efficacy and safety of PFA and TA (radiofrequency and cryoballoon). Pulsed field ablation was associated with a shorter procedure time but longer fluoroscopy time than TA. In terms of efficacy, PFA demonstrated a better first‐pass isolation rate and lower treatment failure rate. Regarding safety, PFA showed lower rates of esophageal injury but higher rates of tamponade. They discussed potential reasons for the higher tamponade rate after PFA, including the initial use of an exceptionally rigid guidewire for PFA catheter delivery, leading to inadvertent left atrial appendage perforation in four patients. Another factor was the tight locking mechanism between the dilator and sheath, which could cause sudden unintended forward motion of the sheath. These issues stem from device preparation and are relatively easy to improve. Rudolph et al.^5^ published another meta‐analysis comparing PFA with cryoballoon ablation for AF treatment. They included data from 11 studies involving 3805 patients. PVI by PFA was associated with significantly lower recurrence and fewer periprocedural complications. In this analysis as well, the lower complication rate with PFA was primarily due to fewer phrenic nerve injuries, although there were more instances of cardiac tamponade following PFA.

Despite the current lesser experience with PFA compared to TA, multiple meta‐analyses have consistently shown benefits such as shorter procedure times, lower rates of arrhythmia recurrence, and fewer complications. However, the initial higher incidence of cardiac tamponade with PFA warrants careful attention. Operators need to be particularly mindful of the elevated incidence of cardiac tamponade during the initial learning curve phase. Furthermore, most studies included in these meta‐analyses are nonrandomized, potentially introducing biases in patient demographics and characteristics. To establish the true efficacy and safety of this promising new energy source, PFA, more high‐quality studies, including randomized controlled trials comparing PFA with TA, are essential and should be prioritized for future research and evaluation.

## FUNDING INFORMATION

Dr. Kuroki (KK) reports grants from Medtronic Co., Ltd. and Abbott Medical Japan LLC. Dr. Kuroki (KK) is a consultant of Abbott Medical Japan LLC., Microport CRM Japan Co., Ltd, and Kaneka Corporation. Sato (AS) reports grants from Abbott Medical Japan LLC.

## CONFLICT OF INTEREST STATEMENT

Authors declare no conflict of interests for this article.
